# Structuring colloidal gels *via* micro-bubble oscillations

**DOI:** 10.1039/d2sm01450e

**Published:** 2023-03-13

**Authors:** K. W. Torre, J. de Graaf

**Affiliations:** a Institute for Theoretical Physics, Center for Extreme Matter and Emergent Phenomena, Utrecht University, Princetonplein 5 3584 CC Utrecht The Netherlands k.w.torre@uu.nl

## Abstract

Locally (re)structuring colloidal gels – micron-sized particles forming a connected network with arrested dynamics – can enable precise tuning of the micromechanical and -rheological properties of the system. A recent experimental study [B. Saint-Michel, G. Petekidis, and V. Garbin, *Soft Matter*, 2022, **18**, 2092] showed that local ordering can be rapidly induced by acoustically modulating an embedded microbubble. Here, we perform Brownian dynamics simulations to understand the mechanical effect of an oscillating microbubble on the next-to-bubble structure of the embedding colloidal gel. Our simulations reveal hexagonal-close-packed structures over a range that is comparable to the amplitude of the oscillations. However, we were unable to reproduce the unexpectedly long-ranged modification of the gel structure – dozens of amplitudes – observed in experiment. This suggests including long-ranged effects, such as fluid flow, should be considered in future computational work.

## Abstract 2

Using Brownian dynamics simulations, we show that an oscillating microbubble can locally crystallize an attractive colloidal gel above a threshold frequency. We relate this to the moving interface's ability to extract colloids from the gel network.

## Graphical abstract

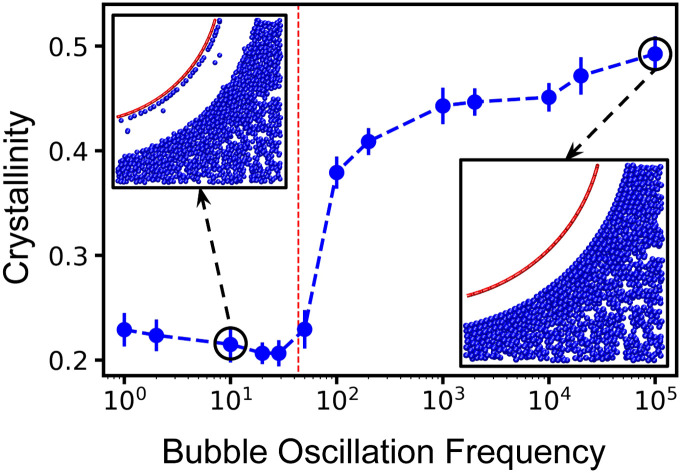

## 1. Introduction

A colloidal suspension can gel, when short-ranged attractions are present with a well depth that greatly exceeds the thermal energy *k*_B_*T*;^1^ here, *k*_B_ is the Boltzmann constant and *T* the temperature. Key to attractive colloidal gelation is the short-ranged nature of the potential well close to the colloid surface. This can be induced by, *e.g.*, the presence of polymers^2^ (depletion) or van der Waals interactions.^3,4^ The depth and short-ranged nature of the well interfere with thermal rearrangement of clustered colloids, thereby arresting the system's natural tendency to fully phase separate. Following a deep quench into the spinodal region of the phase diagram, diffusion-mediated clustering combined with kinetic arrest leads to the formation of an open, space-spanning network structure that is thus intrinsically out of equilibrium.^3,5^ Such a network structure has useful properties, *e.g.*, it can for a finite (often long) time support the gel's buoyant weight against gravity.^6,7^ Stability at low volume fraction, has led to the widespread use of particle gels in industrial, medical, and academic settings, *e.g.*, care products, printing inks, foodstuffs, crop protection, and pharmaceutical suspension formulations.^8–11^ This has led to scientific interest in the properties of colloidal gels, and such systems have been studied using experimental,^6,7,12–21^ computational,^22–33^ and theoretical^1,7,34–39^ methods.

Gels coarsen over time, as the system relaxes toward equilibrium, and their bulk properties can strongly depend on the preparation history,^40^ including oscillatory-shear,^41^ and steady-shear protocols.^42,43^ That is, the preparation can leave a clear signature in the microstructure of the gel,^43^ which expresses itself in the mechanical response of the material.^44^ Modifying a gel's properties *via* external means has mostly focused on the bulk response. However, for many processes, it can be favorable to apply these modifications locally both for colloidal^45^ and other types^46^ of gel.

Recently, Saint-Michel *et al.* showed that a deformable inclusion, taking the form of a (micro)bubble, can be used to locally tune a gel's microstructure.^47^ In their experiments, ultrasound was used to cause the bubble to contract and expand, inducing a periodic strain on the surrounding gel. The study revealed a non-trivial rearrangement of the colloidal network into a crystalline structure. The most interesting feature is the long range – comparable to the bubble radius – over which the rearrangements took place, when only small oscillations (∼1% of the bubble diameter) were employed. The exact physical mechanism behind this long-range rearrangement remains unclear. Locally perturbing the system using ultrasound and gaseous inclusions can also be useful to probe the rheological response at the scale of the microstructure.^48–51^ It is therefore worthwhile to better understand the interaction between inclusions and the embedding colloidal gel.

In this work, we use computer simulations to investigate bubble-oscillation based local reordering of colloidal gels. Our model is based on an effective, Asakura–Oosawa-like description of depletion interactions between the colloids, following an earlier analysis of gelation.^32^ The microbubble is described using a bead-spring model subjected to an external (radial) forcing that models pressure changes, due to the ultrasound. We take into account only the mechanical interactions in our model, *i.e.*, we ignore hydrodynamic interactions between the colloids and porous-medium flow.

For experimentally relevant colloid volume fractions, we vary the colloid-bubble size ratio, the frequency, and amplitude of the oscillations. This allowed us to construct a state diagram that highlights the effect of the oscillations. We find that crystalline reordering into a hexagonal closed-packed state around the bubble is possible under the following two conditions. (i) Multiple layers of colloids need to be compressed by the extensional driving of the bubble. (ii) The frequency of the oscillations is large enough to avoid extracting colloids from the gel network. Turning to the range of the rearrangements, our analysis reveals that this is roughly twice the amplitude of the oscillation. This is a much smaller range than was reported in ref. 47, which suggests that there is a missing ingredient to the modeling. Within the scope of this work, we do not resolve this open problem, however, we do comment on its likely origin, fluid flow, which is not included in our description. The present study lays a solid foundation for future work in this direction.

The rest of this paper is organized as follows. We first introduce our numerical method. Next, we cover how we analyze our results, before we show the state diagram. This is followed by a discussion of the relevant time scales and an outlook on follow-up studies.

## 2. Numerical method

We want to study the influence of an oscillating microbubble on the microstructure of a colloidal gel. We do so by performing Brownian dynamics simulations. These take into account the friction between colloids and solvent at a one-body level, *i.e.*, the particles experience Stokes drag, ignoring any two- or many-body interactions. We also ignore any flows in the gel network caused by the motion of the gas–liquid interface and colloids.

Under these assumptions, the overdamped equations of motion for a single colloid in our system read:1
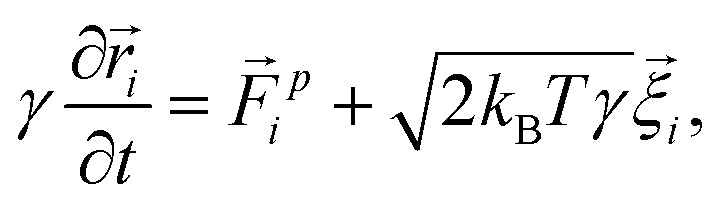
with *r⃑*_*i*_ the *i*th colloid's position. The prefactor *γ* = 3π*ησ* specifies the fluid friction experienced by a single particle, assuming here the Stokes form for a sphere with *η* the viscosity. The force *F⃑*^*p*^_*i*_ acting on the *i*th colloid, derives from pair interactions with neighboring colloids *via* the potentials specified below. The term *ξ⃗*_*i*_ is a random vector that accounts for thermal fluctuations, which are independent and have a white-noise spectrum. That is, we ensure a zero mean 〈*ξ⃗*_*i*_(*t*)〉 = 0⃑ – the angled brackets indicate a time average – and 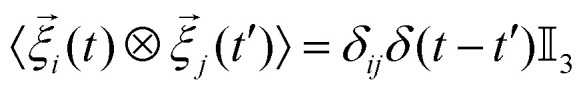. Here, ⊗ indicates the tensor product, *δ*_*ij*_ represents the Kronecker delta, *δ*(*t* − *t*′) the Dirac delta, and 
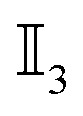
 is the 3D identity matrix.

**Fig. 1:**
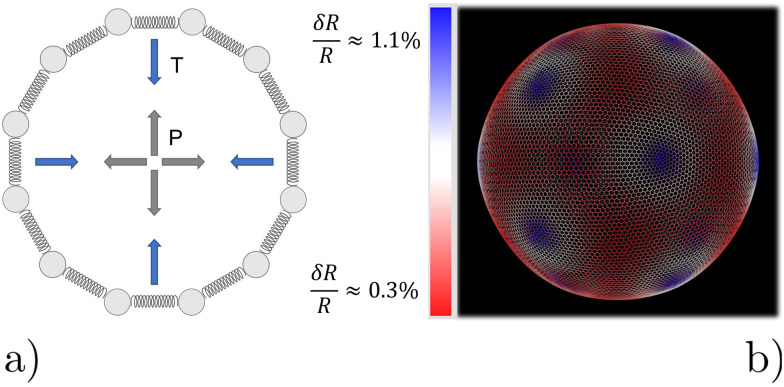
Model for the oscillating microbubble. (a) Two-dimensional sketch of the bead-spring model. Radially outward ‘pressure’ forces (gray) counterbalance inward contraction from tangential springs (blue) representing ‘surface tension’. (b) Snapshot of one of our geodesic spheres at rest. The *very* small non-uniformity of the radius induced by point defects, expressed as δ*R*, is highlighted using the colors in the legend. Blue areas (centered around the 12 pentagonal defects) have a slightly larger radius than the mean value 〈*R*〉, while red areas have a smaller radius.

We model the microbubble as a collection of points that define a geodesic polyhedron. The facets spanned by the vertices represent the bubble surface. We emulate the internal pressure by adding a constant outward-pointing force acting on each vertex. Surface tension is modelled by connecting neighboring vertices *via* harmonic springs. The spring constant and equilibrium pressure are tuned in such a way that the bubble has a mean radius of 〈*R*〉 at rest. The energy scale associated with the spring constant *k*_s_ = 10^7^*k*_B_*Tσ*^−2^, where *σ* is the colloid diameter, and we used an equilibrium pressure *p*_0_ = *Ωk*_s_/4π〈*R*〉, with *Ω* ∈ [0.22,3.14]10^−3^ a dimensionfree coefficient given by the bubble tessellation. Our choices ensure that when the bubble oscillates, it forces the gel out of the way sufficiently vigorously not to cause distortions in its (nearly) spherical shape; in line with the experimental observations. The resulting model bubble is represented in Fig. 1.

We had to account for topological constraints imposed by working with a spherical surface, namely that it cannot be tessellated with hexagonal tiles only, 12 pentagonal defects must be present.^52^ These defects introduce distortions away from perfectly spherical in our bubble surface, as can be appreciated from the coloring in Fig. 1. Red denotes a depression in the bubble surface with respect to its mean radius, whilst blue indicates an increase of the radius. The effect is exaggerated in our representation, as the deviations are typically less than 1%. In constructing our geodesic sphere, we have ensured that the defects are located on the vertices of an icosahedron. This localization is convenient, as it allows us to take slices between the defects, where the change in curvature is minimal. In total there are six such slices possible, which proved sufficient to perform a quantitative analysis of the gel, to which we will return to shortly.

We modeled the gel according to the methods detailed in ref. 32. In brief, we simulate only the colloids and account for the presence of the polymers that cause depletion attraction *via* a generalized “high-exponent” Lennard-Jones potential2
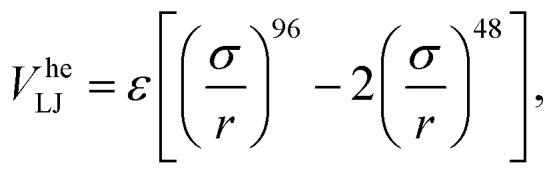
where *r* the center-to-center distance, and *ε* the interaction strength is set to 20*k*_B_*T*. This is a smooth approximation of the well-known Asakura–Oosawa interaction^53^ in combination with steric repulsion. The beads comprising the interface (*i.e.*, of our bead-spring bubble) can interact with the colloids forming the gel *via* the same potential *V*^he^_LJ_ with one modification. The interaction strength of the bead-colloid potential is appropriately rescaled to reproduce the effective depletion interaction between a colloid and the bubble surface. This is roughly twice that present between the colloids themselves; using a flat-wall approximation.††This can be straightforwardly computed from the overlap between a sphere and a wall in the Asakura–Oosawa formalism. We verified that attractions between the bubble and colloids do not play a role in the high-frequency restructuring regime.

Our simulations were performed in periodic, cubic boxes with an edge length *L* ∈ [50*σ*, 120*σ*]. We have chosen system sizes such that periodicity does not influence our measurements. That is, the bubble does not interact with itself, not even *via* the perturbed gel medium. In each simulation, we used a volume fraction *ϕ* = 0.44. This is a rather high value for colloidal gelation, but was chosen to closely approximate that of the experiment.^47^ The bubble radius at rest was chosen to be *R*_0_ ≡ 〈*R*〉 ∈ [10*σ*, 40*σ*]. This choice departs from the value of the experiment – the ratio of colloid-to-bubble radius therein is ≈10^2^ – but proved necessary to achieve a desired computational efficiency. In experiment,^47^ the curvature of the bubble is therefore lower than in our simulations, and the colloids near the interface thus interact with an almost flat surface. We will return to the consequences of this choice in our discussion.

**Fig. 2:**
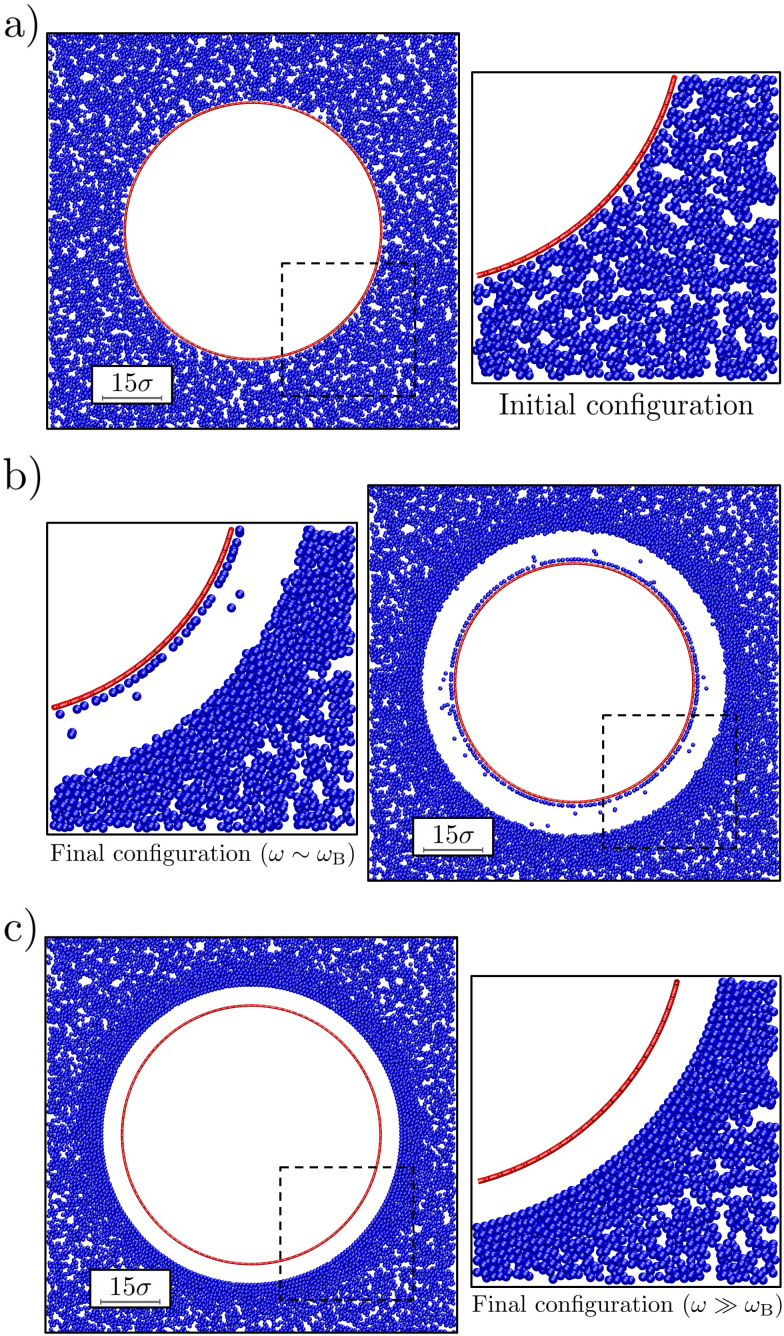
The effect of bubble oscillations on the surrounding colloidal gel. The bubble radius at rest *R*_0_ ≈ 30*σ* and the oscillation amplitude Δ*R* ≈ 4*σ*. Each panel shows a representative snapshot of the system. The main element is a vertical slice through the system that avoids the defects in the geodesic sphere (red circle) and has a width of 2*σ*. This is complemented by a zoom-in (dashed square) on the region where the colloidal gel (blue) is most strongly distorted. Panel (a) shows the starting configuration, while (b and c) show steady-state configurations (after 50*τ*_B_) for values of frequency *ω* = 10*ω*_B_ and 10^5^*ω*_B_, respectively.

The gel was prepared *via* an instantaneous deep quench from a purely repulsive potential to one with the aforementioned 20*k*_B_*T* attraction strength. We allowed the gel to form for 50*τ*_B_, where *τ*_B_ = *σ*^2^/(4*D*) is the Brownian time of the colloids with single-particle translational diffusion coefficient *D*. During this time, the bubble was left unperturbed, in order to allow the system form the gel network and relax internal stresses. This resulted in a gel with an average contact number of 7.4 and void volume of 0.11*σ*^3^ in the bulk, *i.e.*, away from the bubble surface, as measured using the methods of ref. 32,43,54. Fig. 2a shows a representative snapshot of the initial configuration. After preparation of the bubble-gel system, the bubbles, were made to oscillate for 50 cycles, which proved sufficient to obtain steady-states, with different values of the frequency *ω* ∈ [*ω*_B_, 10^5^*ω*_B_] and the oscillation amplitude Δ*R* ∈ [*σ*, 5*σ*]; here we have defined a Brownian frequency *ω*_B_ = (2π/*τ*_B_). The oscillations were induced applying a sinusoidal perturbation on top of the equilibrium bubble pressure *p*(*t*) = *p*_0_(1 + *δ *sin* ωt*). All simulations were performed using HOOMD-blue, a GPU-compatible Python package developed in the Glotzer Lab.^55^

## 3. Characterization

We observed that our model bubble's motion modified the structure of the surrounding gel as follows. Fig. 2 shows a representative snapshot of the initial and steady-state configurations that we obtained for small and large angular (oscillation) frequencies *ω* compared to the Brownian frequency; oscillation amplitude Δ*R* = 4*σ*. In both cases, a void was formed between the gel and the bubble (at rest), which in experiment would be filled with fluid. Further out from the bubble, the colloid density visibly increased. At the largest distances the gel network appeared unperturbed. For *ω* ∼ *ω*_B_ the denser region appears disordered (Fig. 2b), while for *ω* ≫ *ω*_B_ (Fig. 2c) the dense region is clearly ordered.

For the lowest applied frequencies, we even observe rupture of the gel network, as evidenced by a layer of colloids that have detached from the network, but are still bound to the bubble surface *via* the depletion interaction. Also note that some of the colloids have become detached from both the surface and the gel, and are freely floating in the ‘fluid-filled’ void between the bubble surface and the gel in Fig. 2b. The results presented in Fig. 2 suggest a connection between frequency of oscillation and local reordering of the colloidal gel close to the bubble. We quantified this using averaged local bond-order parameters.^56^ These are non-dimensional parameters that can be used to distinguish ordered structures from disordered ones. In particular, we choose *q*_6_ as indicator of reordering in the system, as it is the most significantly affected by the bubble oscillations.

**Fig. 3:**
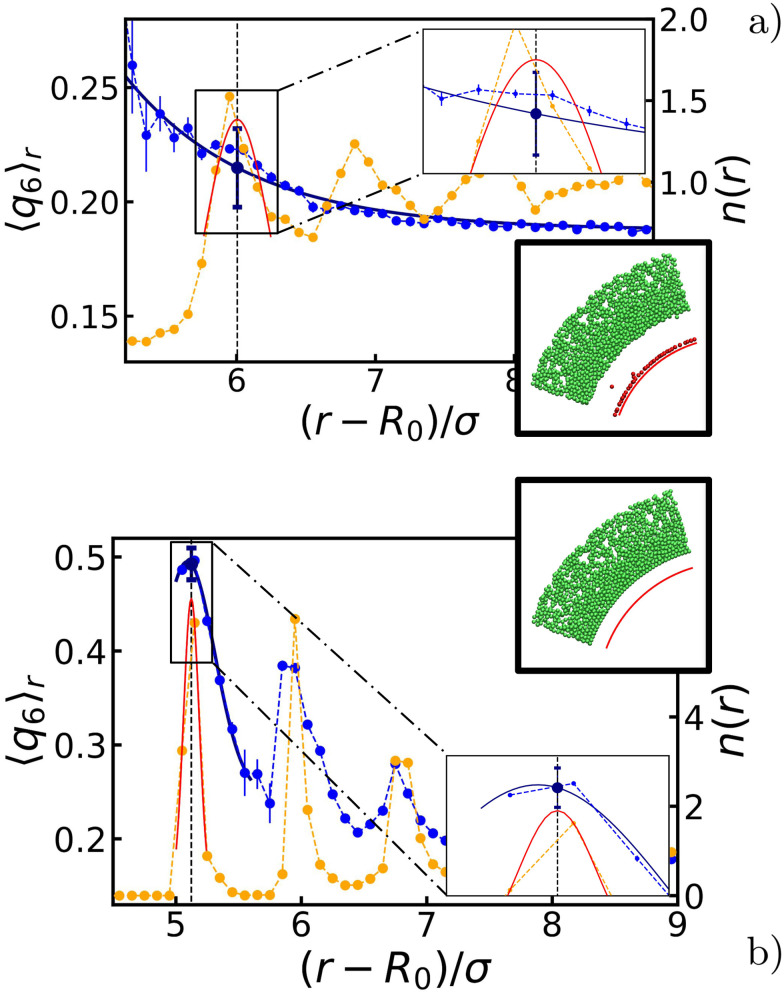
Quantization of the ordering effect of the bubble oscillations on the surrounding gel. The radial density function *n*(*r*) (yellow) and the radially averaged *q*_6_ bond-order parameter 〈*q*_6_〉_*r*_ (blue). The symbols provide our data points and the error bars indicate the standard error of the mean. The blue and red fitted curves are used in our analysis procedure, as described in the main text. Through the fit procedure, we locate the position of the first peak in *n*(*r*), as indicated using the vertical gray dashed line. The second set of insets shows a wedge from a snapshot taken at steady state. Green particles remained part of the gel network, while red particles were either in a gas phase or has become attached to the bubble surface (represented here by the red arc). Detached particles are not considered in our analysis, as they do not contribute to the gel microstructure. Panels (a and b) show the steady-state profiles for a bubble-oscillation angular frequency of *ω* = 10*ω*_B_ and *ω* = 10^5^*ω*_B_, respectively. For both (a and b) the bubble radius at rest is *R*_0_ ≈ 30*σ* and the oscillation amplitude is Δ*R* ≈ 4*σ*.

Given the symmetry of the system, we made use of a radial average 〈*q*_6_〉_*r*_, *i.e.*, we measured the quantity shell by shell. The effect is pronounced around those layers that are (in temporary) contact with the bubble, and we therefore focused on the first few intact particle shells, as measured from the center. Two representative results are shown in Fig. 3. Comparing the values before and after the oscillations, we choose to represent each configuration with a single 〈*q*_6_〉 value taken at distance r* and denoted henceforth by 〈*q*_6_〉_r*_. Here, r* is the value, at which the radial density function *n*(*r*) has its first peak and the radially averaged coordination number 〈*z*〉_r*_ ≥ 6. The introduction of 〈*q*_6_〉_r*_ will prove useful in constructing our state diagrams.

Appendix A provides the details of our procedure to arrive at 〈*q*_6_〉_r*_. In brief, we fitted each peak to *n*(*r*) using a Gaussian function to determine r*. For 〈*q*_6_〉_*r*_, we instead used a decaying exponential for disordered configurations (Fig. 2b), and a Gaussian function for ordered ones (Fig. 2c). We computed the standard error of the mean by summing uncertainties in the data and variances from the fitted functions, with the former being typically negligible compared to the latter.

Lastly, we quantified the length scale associated with the ordering in the system by fitting the peaks in 〈*q*_6_〉_*r*_ (when present) together with the values in the bulk. We found that the extent of the crystal structures is roughly double the amplitude of oscillations, *i.e.*, 2Δ*R*. The derivation of this typical length and the detail of the fits are provided in Appendix A.

## 4. State diagram

**Fig. 4:**
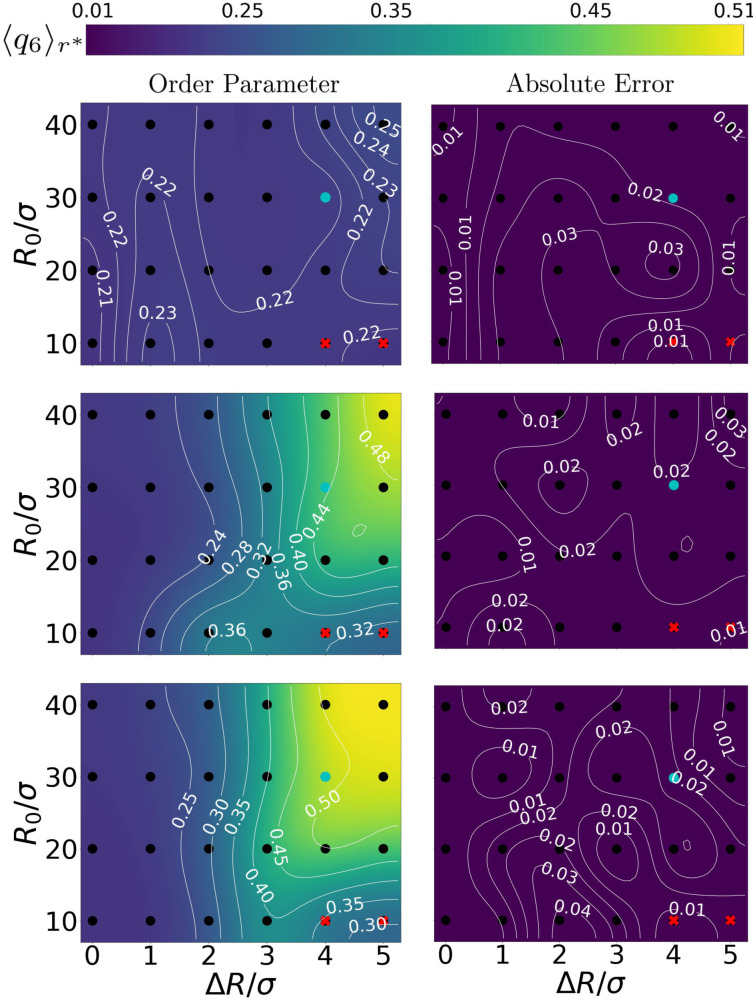
Charting the effect of bubble radius *R*_0_ and oscillation amplitude Δ*R* on the ordering of the gel. The panels come in pairs (a) and (b), *etc.*, and respectively show (left) the value of 6-fold, bond-order parameter in the first shell 〈*q*_6_〉_r*_ and (right) the absolute error therein. From top to bottom the angular frequencies of the bubble oscillations are *ω* = 10^1^*ω*_B_, 10^3^*ω*_B_, and 10^5^*ω*_B_, respectively. The points where we obtained our data, are indicated using (black) dots. A smooth interpolation (color map) was created using a bicubic scheme,^57^ in which new grid-points with associated function values are iteratively computed from four neighbouring points. The thin white curves provide iso-value contours. Data points marked with a red cross are excluded from our analysis because they belong to regions that are biased by the bubble tessellation, as explained in the main text. The cyan points represent configurations that are analysed in Fig. 5.

The above quantitative analysis allowed us to map the explored configurations onto a state diagram. Fig. 4 shows three such mappings, providing the enhancement in local order using the steady-state 〈*q*_6_〉_r*_ as a function of the bubble radius *R*_0_ and oscillation amplitude Δ*R* (both normalized by the colloid diameter *σ*). We show the result for a low, an intermediate, and a large *ω* compared to *ω*_B_. We confirm the absence of constructive (increasing *q*_6_) restructuring – 〈*q*_6_〉_r*_ is left nearly unperturbed – for the low-frequency configurations *ω* = 10*ω*_B_. For intermediate values of frequency *ω* = 10^3^*ω*_B_, we see the emergence of a wide region in the phase diagram where 〈*q*_6_〉_r*_ reaches values that are slightly over twice (≈0.5) those of the initial configurations (≈0.2). The highest frequency regime shows similar features, but the zone of ordering in the state diagram is appreciably wider. These findings further support the idea that local crystallization of the gel is strongly controlled by *ω*.

The data in Fig. 4 allows us to conclude that ordering is triggered only for sufficiently large bubbles (*R*_0_ ≥ 20*σ*). Additionally, inducing local order in the gel is only possible, when the bubble can sufficiently expand and contract the surrounding gel. As there are no prescribed long-ranged interactions in our simulations (no hydrodynamic flows), compression in the gel is entirely dictated by the oscillation amplitude and mechanical propagation. We find that ordered structures can only emerge for Δ*R* ≳ 3*σ*, *i.e.*, amplitude plays a minor role.

Here, we should note that there are small areas in Fig. 4 corresponding to small *R*_0_/*σ*, yet large Δ*R*/*σ*, that appear not to be affected by the bubble motion. This is an artifact of our bubble model: the surface discretization becomes comparable to the colloid–colloid separation. This gives rise to an effective egg-carton-like potential (for large values of Δ*R*/*R*_0_) that induces preferential colloid positions at the interface, which would not be present for a molecular interface. This effective bubble-colloid interaction interferes with structuring.

Avoiding these artifacts, we realize that *R*_0_/*σ* ratio determines the geometry of the collision between the gel network and the expanding bubble. For *R*_0_ ≫ *σ*, the network experiences an interaction with an almost flat surface. This favors the alignment of the colloids in the gel, promoting the formation of ordered structures.

## 5. Oscillation frequency

**Fig. 5:**
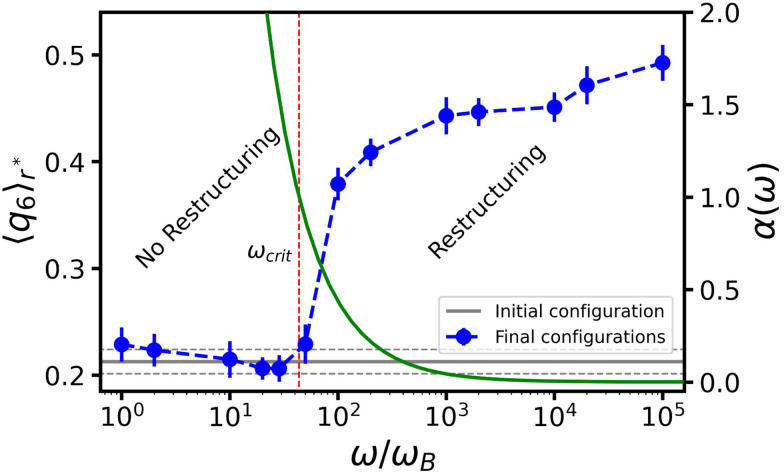
The effect of angular frequency *ω* on the ordering of the gel around an oscillating bubble. Here, we choose *R*_0_ = 30*σ* and set the oscillation amplitude at Δ*R* = 4*σ*. Blue circles provide the peak value of the bond-order parameter 〈*q*_6_〉_r*_, given as a function of *ω* normalized by the Brownian frequency *ω*_B_. The error bars indicate the standard errors of the mean. The horizontal gray line indicates the 〈*q*_6_〉_r*_ value of the initial configuration (before the bubble oscillations) and the two dashed lines the associated standard error of the mean. The green curve represents the dimensionfree parameter *α* as a function of *ω* – values indicated on the right-hand *y*-axis – and the red dashed vertical line indicates the frequency, at which we locate the crossover between two regimes (*α* = 1); the main text provides the definition.

We will focus on the large *R*, high *ω* situation next, as it more closely aligns with the experimental setup of ref. 47. By fixing *R*_0_ = 30*σ* and Δ*R* = 4*σ*, we can isolate the effect of the angular frequency *ω*, see Fig. 5. We observe two trends in 〈*q*_6_〉_r*_ as a function of *ω*, which are separated by a relatively sharp transition. For small values of *ω* there is no change in the order, while for sufficiently large *ω*, 〈*q*_6_〉_r*_ saturates to its crystalline value. This aligns with our analysis in Fig. 4. Fig. 5 suggests that reordering in the gel is possible only if the time scale associated with bubble motion is negligible compared to thermal diffusion of the colloids.

To understand the role of *ω*, we make an analogy to the frequency response of a colloidal gel under oscillatory shear.^58^ Using a Kramer's argument, the authors of ref. 58 estimated the effect of shear on the probability for a particle to escape from the attractive potential of its neighbor. Considering the typical escape time as a function of the shear frequency, they concluded that there is a critical threshold, above which the particles can rearrange (to form crystalline structures).

Motivated by this, we contrast the period of oscillation with two times scales in our system: the thermal diffusion time and a network-extraction time, respectively. The former is *τ*_B_ ≥ 2π*ω*^−1^ in all our simulations (*ω* ≥ *ω*_B_ in Fig. 5) and we therefore deem it irrelevant. For the latter, we obtain the dimensionfree combination3
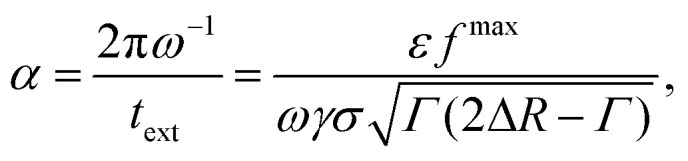
see Appendix B for a detailed derivation, where4
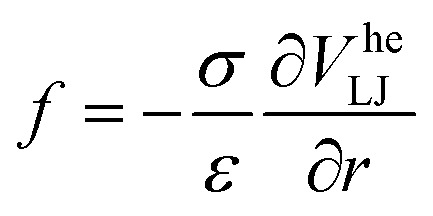
is the dimensionfree inter-particle force and *f*^max^ the absolute of its minimum value; *Γ* indicates the width of the potential well. We estimate the network-extraction time *t*_ext_ by comparing the velocities of the bubble and a single colloid attached to its surface. Fig. 5 shows that *α* = 1 is a natural bound between a regime of negligible and pronounced ordering, respectively.

We first turn to the regime in which there is no constructive reordering (*α* ≥ 1; *ω* ≲ 10^2^*ω*_B_), see Fig. 5. Here, the bubble shrinks sufficiently slowly to allow colloids to be extracted from the gel for each contractive part of the oscillation cycle. The consequence is that branches of the gel network near the bubble surface are continuously ripped apart and reformed. This impedes the formation of crystalline layers. For the lowest frequencies applied in our simulations, we even observed the formation of a monolayer of colloids attached to the bubble surface. In our modeling they are attached *via* depletion, but in experiment wetting could play a strong role as well.

For moderate and high frequencies (*α* < 1; *ω* ≳ 10^2^*ω*_B_), the bubble moved too fast to extract particles from the surrounding gel. As a result, the colloids in the network only experience a force pointing away from the bubble surface, which slowly pushes the surrounding gel outward. This ultimately leads to the formation of a zone wherein the gel is locally compressed; the gel further away from the bubble surface resists the radial expansion. The combined effect of local compression and oscillatory perturbations allows the colloids to overcome their kinetic barriers and rearrange into a (locally) crystalline structure. We conclude that *α* is a meaningful parameter and that structuring is predominantly controlled by the bubble's inability to extract colloids from the network, as this interferes with the process of crystallization.

## 6. Discussion

Our simulation results align with the experiments of ref. 47, in the sense that local tuning of the microstructure can be triggered by oscillations of deformable inclusions. Above, we have only considered a single volume fraction *ϕ* = 0.44, as was used in experiment. However, in many gel-based applications, lower values of *ϕ* are used. We performed several additional simulations (not shown here) at high frequency *ω* = 10^5^*ω*_B_ for *ϕ* = 0.2 and *ϕ* = 0.3 to verify the robustness of our results. The effect of lowering *ϕ* (*R*_0_ = 30*σ* and Δ*R* = 4*σ*) is a slight lowering of the value of 〈*q*_6_〉_r*_, by at most 20%. This is a direct consequence of the lower colloid volume fraction around the bubble, leading to weaker compaction of the gel by equally strong oscillations. Inducing crystallization at lower *ϕ* will require greater amplitudes.

Despite our ability to observe local structure formation, there remains a qualitative mismatch between simulation and experiment. In the latter, ordering was observed over a radial range that significantly exceeded the amplitude of oscillation. Additionally, in our simulations we observe the formation of a pocket of solvent around the oscillating bubble. In experiment, a solvent pocket was only found as a consequence of bubble dissolution; a process that we do not model. Lastly, in our simulation there is a colloid density heterogeneity following bubble oscillation, which appears absent in the experiment.

We consider it possible that the missing ingredient to have long-ranged structuring without density heterogeneities, is fluid flow. The porous-medium flow produced by the oscillating bubble, has the potential to influence particles that are far away from the surface. Provided a sufficiently large shear-Péclet number can be achieved,^58^ bond-breaking rearrangements can then be made further out from the bubble. Alternatively, the time-varying flows can serve to raise the effective temperature of the colloids in the gel network, thereby facilitating rearrangement. In either scenario, the reduced need to push the colloids, could help reduce density heterogeneities, while still increasing the local order.

Including porous medium flow in simulation could allow crystal structures to emerge at distances greater than we predict in our Brownian-dynamics study. Additionally, this flow would also allow colloids to be dragged back and forth during the oscillations, potentially avoiding the creation of strong density gradient around the bubble, as we observed in our study, but which appears not to be present in experiment. However, this is a hypothesis, which should be tested in simulation.

The main difficulty in performing such simulations is the presence of a (moving) gas–liquid interface. Accounting for complex interfaces with large differences in viscosity is a challenge in computational fluid dynamics.^59^ An approximate method to account for the interface would be to ignore the density and viscosity differences and use the presence of the tessellation points on the bubble surface to induce flows. This can, for instance, be done in the Rotne–Prager–Yamakawa (RPY) formalism *via* the HOOMD-blue plugin developed by Fiore *et al.*^60^ Such an approximation relies on the idea that the flows internal to the gel, rather than the presence of a gas–liquid interface control the physics of the rearrangement. The downside of this route is, however, that this is an uncontrolled approximation to the full hydrodynamic problem. That is, there is no means by which to readily refine it through the addition of higher-order terms.

## 7. Conclusions and outlook

Summarizing, we have quantified how an oscillating microbubble embedded in an attractive colloidal gel locally modifies the structure of the gel around its surface using Brownian-dynamics simulations. The effect of the bubble dynamics can be constructive – meaning that the gel locally crystallizes – if the oscillation amplitude and the colloid-bubble size ratio are sufficiently large. The former controls the amount of compression exerted on the gel, and the latter determines the geometry of colloid-bubble collisions. Reordering is observed only in configurations where at least three layers of colloids are compressed, and where the colloids in the gel interact with an almost flat bubble surface.

Beyond these basic requirements, we find that the frequency of oscillation is the control parameter for creating order. The bubble's oscillatory dynamics compete with both thermal- and potential-energy based rearrangements in the system. Crystalline layers are formed only when the oscillations are fast enough not to break up clusters of colloids or extract colloids from the gel network. When there is no destructive rearrangement, the main effect of the oscillations is a slow compression of the surrounding gel network. Local reordering typically extends into the bulk of the gel for a range equal to the oscillation amplitude.

Lastly, the present work lays a solid foundation for understanding the impact of bubble oscillations on gel microstructure. To address the present mismatch between experiment and simulation, in terms of the range of the ordering effect, it would be interesting to consider simulations that properly account for hydrodynamic flow, though this poses a challenging prospect.

## Data availability

Open data package containing the means to reproduce the results of the simulations available at: [https://doi.org/10.24416/UU01-GT8YQ5].

## Conflicts of interest

The authors declare that there are no conflicts of interest.

## Supplementary Material

## Figures and Tables

**Fig. 6:**
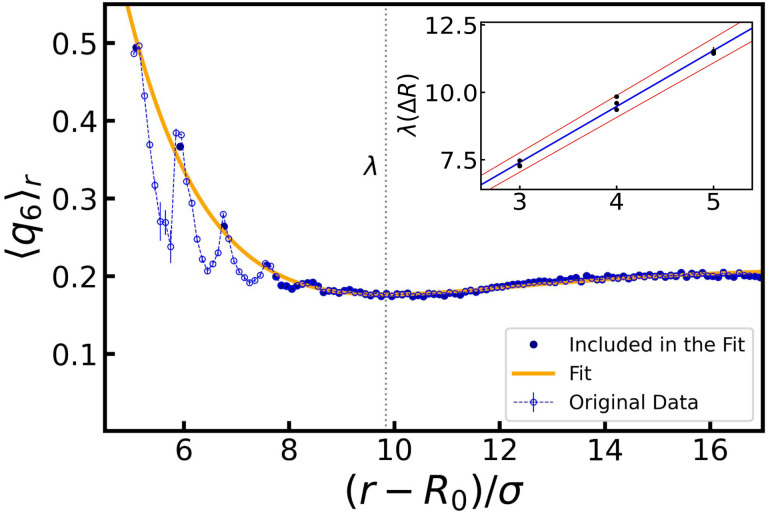
Characteristic length *λ* over which the bubble oscillations reorder the surrounding gel. The original data set for 〈*q*_6_〉_*r*_ (blue circles); *R*_0_ = 30*σ*, Δ*R* = 4*σ*, and *ω* = 10^5^*ω*_B_. The fitted function (orange curve) is computed using selected data from the original data set (highlighted in dark blue): the first Δ*R* peaks, obtained using Gaussian fits, as well as the bulk value of 〈*q*_6_〉_*r*_. The vertical dashed line indicates the position of the minimum of the fit, at which we place *λ*. The inset shows the linear trend – the central value in blue, standard error of the mean delimited by the red lines – obtained from all values of *λ* (9 in total).

## Appendices

### A Fit parameters and justification

In this appendix, we provide the details for various fitting procedures that we employed. We start by describing the procedure used to fit the radial density function *n*(*r*), see Fig. 3. For each configuration studied, we use a Gaussian function to fit the data points in the vicinity of the first peak and estimate the peak position *r**:

5 *n*(*r*) = *a*e^−(*r*−*r**)^2^/2*b*^2^^ + *n*_0_,

with *a*, *b*, and *n*_0_ fitting parameters that are not relevant to the analysis. If the first peak corresponds to a radial shell with an average coordination number smaller than 6, the peak is ignored, as it corresponds to either colloids attached to the bubble or in the gas phase (colloids floating in the ‘fluid-filled’ void between the bubble surface and the gel). In those cases, the second peak is considered; the analysis is otherwise unaffected.

Turning to the peak value of the 6-fold bond-order parameter, 〈*q*_6_〉_*r**_, we include only data points in the vicinity of *r** in our fit of 〈*q*_6_〉_*r*_. We use either an exponential function, if the configuration considered does not show reordering (see Fig. 3a), or again a Gaussian function, in the case that crystalline structures are present in the system (see Fig. 3b):

6

where the constants *a*_*i*_, *b*_*i*_, *c*_*i*_, and *d* are fitting parameters. Subsequently, we evaluate 〈*q*_6_〉_*r*_ for *r* = *r**, thereby obtaining a value that we deem representative of the amount of ordering present in the dense layer.

We obtained an estimate of the length scale over which the bubble oscillations modify the gel structure, by fitting 〈*q*_6_〉_*r*_ for each ordered configuration (*R*_0_ ≥ 20*σ*, Δ*R* ≥ 3*σ*), combining linear and exponential functions:

7 〈*q*_6_〉_*r*_ = *a*(*r* − *κ*)e^−*ξr*^ + *d*.

From the fit parameters, we can extract a characteristic length *λ* = 1/*ξ* + *κ* corresponding to the distance where 〈*q*_6_〉_*r*_ has its minimum, see Fig. 6. In this figure, we focus on configurations with the highest value of the frequency of oscillation (*ω* = 10^5^*ω*_B_), as these show the strongest crystallization effects.

The inset shows the dependency of *λ* on Δ*R*. A linear fit of the obtained values provides us with *λ*(Δ*R*) = *β*Δ*R* + *ν*, where *ν* = 1.2 ± 0.3 and *β* = 2.07 ± 0.07. Here, *β* indicates that when Δ*R* layers of colloids are crystallized, the next Δ*R* layers in the gel have reduced ordering, before saturating to the bulk structure. The value of *ν* indicates that reordering of a single radius of colloids is to be expected, even without oscillations.

### B Extraction time model

In this appendix, we provide a derivation of the network-extraction time, used to define the dimensionfree parameter *α* in eqn (3). We consider a 1D model comprising of the bubble and a single colloid interacting with it. Neglecting thermal forces, their velocities are

8

9

where *r* is the colloid position, *R*_B_ is the bubble radius, and *f* is the dimensionfree force, in our case *f* = 96(Δ*r*/*σ*)^49^[(Δ*r*/*σ*)^49^ − 1], with Δ*r* the center-to-surface distance between the colloid and the bubble.

We want to compare the amplitudes of these velocities for *t* ∈ [*P*/4,*P*/2], which is the interval of time in which the bubble performs a contraction. When the colloid velocity, induced by the depletion forces, is greater than the rate at which the bubble shrinks, then the latter can move the colloid from its original configuration. The condition required is:

10

To simplify the expression we can consider that the left-hand-side has a maximum in Δ*r** ≈ 1.0143*σ*. That is, if the colloid does not keep up with the bubble motion for Δ*r**, it will not be able to do so for any other separation. Moreover, if the colloid has not yet been extracted, when the bubble leaves the potential well, it will be unlikely for it to do so at later times. We then restrict our analysis to *t* ∈ [*P*/2,*P*/2 + *t̃*], where *t̃* is such that *R*_*B*_(*t̃*) = *R*_0_ + Δ*R* − *Γ* and *Γ* is the width of the potential well. By maximizing the right-hand-side of inequality 10 with respect to time and computing the left and side for Δ*r* = Δ*r**, the inequality can be reassembled as

11

and define the extraction time. Thus, we predict that for oscillation periods greater than *t*_ext_, the colloids can be extracted from the network at each cycle of the bubble oscillations, preventing order to form.
